# Effect of weighting for sampling and non-response on estimates of STI prevalence in the third British National Survey of Sexual Attitudes and Lifestyles (Natsal-3)

**DOI:** 10.1136/sextrans-2019-054342

**Published:** 2020-03-27

**Authors:** Guy Harling, Andrew Copas, Soazig Clifton, Anne M Johnson, Nigel Field, Pam Sonnenberg, Catherine H Mercer

**Affiliations:** 1 Institute for Global Health, University College London, London, UK; 2 Africa Health Research Institute, KwaZulu-Natal, South Africa; 3 MRC/Wits Rural Public Health & Health Transitions Research Unit (Agincourt), University of the Witwatersrand, Johannesburg, South Africa; 4 Department of Epidemiology & Harvard Center for Population and Development Studies, Harvard T.H. Chan School of Public Health, Boston, MA, United States; 5 NatCen Social Research, London, UK

**Keywords:** bacterial infection, epidemiology (general), statistics, HPV, HIV

## Abstract

**Objectives:**

In addition to researcher-designed sampling biases, population-representative surveys for biomarker measurement of STIs often have substantial missingness due to non-contact, non-consent and other study-implementation issues. STI prevalence estimates may be biased if this missingness is related to STI risk. We investigated how accounting for sampling, interview non-response and non-provision of biological samples affects prevalence estimates in the third National Survey of Sexual Attitudes and Lifestyles (Natsal-3).

**Methods:**

Natsal-3 was a multistage, clustered and stratified probability sample of 16–74 year-olds conducted between 2010 and 2012. Individuals were sampled from all private residential addresses in Britain; respondents aged 16–44 were further sampled to provide a urine specimen based on characteristics including self-reported sexual behaviours. We generated prevalence estimates and confidence intervals for six STIs in five stages: first without accounting for sampling or non-response, then applying inverse-probability weights cumulatively accounting for interview sampling, interview non-response, urine sampling and urine non-response.

**Results:**

Interview non-completion occurred for 42.3% of interview-sampled individuals; urine non-completion occurred for 43.5% of urine-sampled individuals. Interview-sampled individuals, interview respondents, those selected for urine samples and those providing urine samples were each in turn slightly more at-risk for most STIs, leading to lower prevalence estimates after incorporating each set of weights. Researcher-controlled sampling had more impact than respondent-controlled response.

**Conclusions:**

Accounting for both sampling structures and willingness to interview or provide urine specimens can affect national STI prevalence estimates. Using both types of weights, as was done in Natsal-3, is important in reporting on population-based biomarker surveys.

## Introduction

Representative samples of individuals with biomarkers for STIs are vital to understanding both the population burden of STIs and monitoring trends.^1^ Biological measures have the benefit over self-reported measures of avoiding social desirability bias and recall error, as well as capturing asymptomatic infections. Population-based surveys also avoid biases in clinic-based surveillance data, for example, STI diagnoses, including differential access to care and under-detection of asymptomatic infection. However, collecting and analysing population-based data is challenging. Such challenges include building a sampling frame from which to draw, non-response due to non-contact (ie, individuals are untraceable), and non-consent (ie, individuals decline to participate) as well as difficulties in collecting and processing biological samples in a non-clinical setting. Unless respondents are missing completely at random (MCAR), unweighted STI prevalence estimates using population-based samples will be biased.

One common method that can overcome some of these biases is inverse-probability weighting (IPW), which can account for under/oversampling of subpopulations, and for some types of sampled individuals being less/more likely to respond.^2^ IPW involves up-weighting responses from under-represented subpopulations and down-weighting those from over-represented groups, so that the final, weighted sample reflects the population from which they were drawn. While these weights can be precisely calculated for intentional sampling, for non-response they must be estimated from available information on non-respondents—which may be limited. As a result, IPW can only account for non-respondents if they are missing at random (MAR), that is, where missingness depends only on observed characteristics, but not if they are missing not at random (MNAR), that is, missingness is a function of unobserved characteristics.

Non-response IPWs are commonly used in representative health studies using biomarkers, including the National Health and Nutrition Examination Survey (NHANES) and Health and Retirement Survey (HRS) in the USA and the English Longitudinal Study of Ageing (ELSA),^3–5^ as well as in the British National Survey of Sexual Attitudes and Lifestyles (Natsal). In this article, we demonstrate how sampling and non-response weighting led to the reported estimates of STI prevalence in the third Natsal survey (Natsal-3).^6^

## Methods

Natsal-3 was a multistage, clustered and stratified probability sample survey of the British resident population, using face-to-face and computer-assisted self-interviews.^7 8^ The survey first selected postcode sectors, then randomly selected households within those sectors based on the Postcode Address File (PAF), a regularly updated list of all private residential addresses nationally, and finally invited a randomly selected eligible household member (aged 16–74) to participate. Interviews were conducted between September 2010 and August 2012. Postcode sectors were a priori stratified by region, population density, age and economic profile; within strata sectors were selected with probability proportional to number of addresses.

Following the interview, a stratified subsample of participants was invited to provide a urine specimen, with undersampling of lower-risk individuals. Natsal-3 sampled all 16–17 year-olds, all 18–24 year-olds reporting any sexual partner ever, all males aged 25–44 reporting having sex with another man in the past 5 years, and a random 85% of other 25–44 year-olds reporting any sexual partner ever. The survey tested urine specimens for *Chlamydia trachomatis*, human papillomavirus (HPV), HIV antibody, *Mycoplasma genitalium*, *Neisseria gonorrhoeae* and *Trichomonas vaginalis*. STI testing was anonymised and results not returned.^6 9^

Natsal-3 aimed to generate nationally representative STI prevalence estimates. IPWs were developed to account for unequal probabilities of sampling, and for non-response, at both the interview and urine provision stages. Interview sampling weights corrected for postcode-level differences in multiple-occupancy addresses, multiple-adult households and for oversampling in London. Interview non-response weights corrected for differences in response by sex, age and region. Urine sampling weights adjusted for the non-random urine sampling outlined above, while urine non-response weights were based on models including a range of sociodemographic and sexual behaviour variables reported at interview found to predict response, specifically: for males—ethnicity, education, marital status, past sexual health clinic attendance, past-year condomless sex partner count, partner concurrency and non-prescription injection drug use; for females—residential region, lifetime partner count, anal sex history, HIV testing history, presence of others during the interview; for both—lifetime report of same-sex sexual experiences. A small number of weight values were trimmed to remove extreme values.^8^

All weights were calculated as the inverse of the probability of participation, that is, IPWs, rescaled to have a mean of one. Weights accounting for more than one type of bias were calculated by multiplying together the weights for each bias, for example, weights for oversampling *and* non-response are calculated as the product of oversampling and non-response weights.

### Statistical analyses

We estimated the prevalence of chlamydia, gonorrhoea, HIV, *M. genitalium*, trichomoniasis and HPV. HPV results are only provided for females given the low sensitivity of HPV tests in urine in men.^10^ For HPV, we estimated prevalence of oncogenic and vaccine-preventable strains, specifically: (1) any oncogenic strain (HPV-16, 18, 31, 33, 35, 39, 45, 51, 52, 56, 58, 59 or 68); (2) nonavalent vaccine-preventable (HPV-6, 11, 16, 18, 31, 33, 45, 52 or 58); (3) quadrivalent vaccine-preventable (HPV-6, 11, 16 or 18); (4) bivalent vaccine-preventable (HPV-16 or 18).^11^ We first estimated the prevalence of each STI without weighting. We then applied IPWs calculated using all applicable weights at each stage: first, interview sampling weights alone; then interview sampling and non-response weights; then both interview weights and urine sampling; then all four weights.

Ethical approval for Natsal-3 was obtained from Oxfordshire Research Ethics Committee A.

## Results

**Figure 1:**
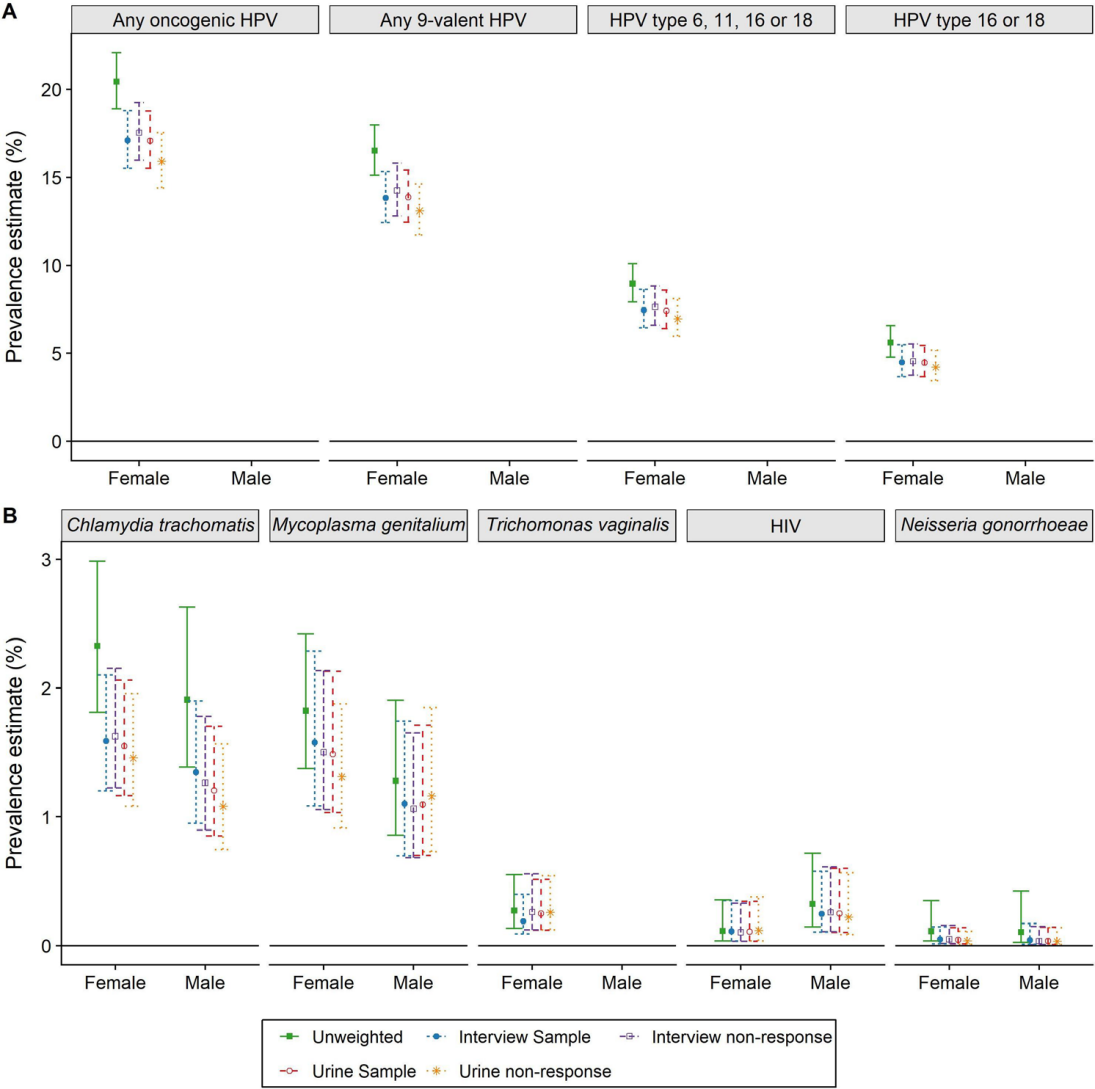
Prevalence of STIs in Natsal-3 adjusted for sampling and non-response weights. (A) HPV; (B) other STIs. All weights are cumulative (eg, interview non-response includes interview sample weights). Precise values are provided in online supplementary table 1. HPV, human papillomavirus; oncogenic HPV are types 16, 18, 31, 33, 35, 39, 45, 51, 52, 56, 58, 59 and 68 (ie, group 1 and group 2A); nonavalent types are 6, 11, 16, 18, 31, 33, 45, 52 and 58. No men tested positive for *T. vaginalis* in Natsal-3.

Natsal-3 sampled 26 274 households. The interview non-response rate, including non-contact and non-consent, was 42.3%. Of the 15 162 interview respondents, 8047 were sampled for urine provision and 4550 (56.5%) provided a sample (online supplementary figure 1).^8^ Prevalence estimates are shown in figure 1. Interview participants were somewhat higher-risk than the general population for all STIs, leading to a drop in estimated prevalence once interview sampling and non-response weights were applied. Most of this drop was due to sampling rather than non-response.

10.1136/sextrans-2019-054342.supp1 Supplementary data

Similarly, those providing a urine specimen were slightly more likely to have each STI tested for (except HIV in women and *M. genitalium* in men where non-providers had higher estimated prevalence). As a result, adjusting for urine sampling and non-response again lowered most prevalence estimates. In all cases, the sampling and non-response effects for urine were in the same direction, and both of similar (small) magnitude. The overall impact of adjustment for sampling and non-response was to lower prevalence estimates from their crude values to the published values containing all four weights,^6^ reflecting both intentional oversampling of, and higher response rates by, higher-risk individuals.

## Discussion

We have highlighted that both sampling and survey non-response can affect point-prevalence estimates in population-based biomarker STI surveys. For most STIs in Natsal-3, the introduction of sampling and non-response weights did not substantially affect prevalence estimates, although researcher-controlled sampling generally had a greater impact than respondent-controlled response. This latter reflects oversampling of higher-risk individuals, based on area-level characteristics at the interview stage and on individual sexual behaviours at the urine provision stage. While interview non-response had a more limited impact on prevalence estimates, accounting for urine non-response did lower some STI prevalence estimates, notably reducing chlamydia prevalence in men by 10% and oncogenic HPV in women by 7% (1.2 percentage points in absolute terms). This reflects participants in Natsal-3 who provided urine having higher-risk characteristics than those who declined.^8^

The relative impact of sampling versus non-response on STI prevalence in population-representative surveys depends on both processes. Non-random sampling with respect to the outcome is often used to improve efficiency and power—interviewing higher-risk individuals increases the number of ‘cases’ identified; the more focused the sampling process, the more impact the resulting reweighting will have on prevalence. Non-response bias depends on the degree to which non-response is a function of the outcome of interest—because higher-risk (or lower risk) individuals cannot be found, do not wish to participate in general, or decline to test for STIs because they believe they are at higher or lower risk of testing positive. In Natsal-3, it appears that the sampling process had more impact than non-response, perhaps because many individuals did not know their STI risk or did not decide to provide urine based on such risk.

While some non-response predictors can be measured and accounted for via IPW (as we do here), others may be unobserved and continue to bias STI estimates. IPWs rely on the untestable assumption that non-responders differ from responders in their STI risk in a manner that can be predicted from observed covariates (ie, MAR). If this untestable assumption does not hold (ie, MNAR), IPW-adjusted figures will be biased. If MNAR is suspected then, under further assumptions, selection models can potentially be used provide more valid estimates both of STI prevalence and CIs.^12^ Alternatively, sensitivity analysis can be used to explore the potential impact of differing strengths of non-response bias on prevalence estimates; this too requires assumptions about plausible levels of bias.

An important potential extension of the use of IPW would be to look at changes in STI prevalence over time. However, such analysis is complex, requiring taking into account differential sampling if using repeated cross-sectional studies, or of differential loss-to-follow-up and learning effects if repeatedly surveying a cohort. In all cases, there is a risk that changes in willingness to participate across waves are associated with STI risk, and any changes in STI test performance over time.^13^ Such biases can be controlled via IPWs insofar as the missingness mechanisms are known, although it is vital to consider whether changes over time may encourage lower-risk or higher-risk individuals to participate differentially based on uncaptured characteristics. In addition, as the precision of estimates of change in STI prevalence will be lower than for the prevalence at one time point, power to detect important change may be restricted.

### Conclusion

Weighting is a standard and necessary part of good survey methodology. This paper demonstrates the quantified contribution of different elements of the weighting strategy to deriving the best estimates of unbiased population prevalence. This was done for Natsal-2 and Natsal-3. It will be important for Natsal-4 as well as other population-based surveys incorporating biological markers.
