# Supplementary material for: Effect of weighting for sampling and non-response on estimates of STI prevalence in the third British National Survey of Sexual Attitudes and Lifestyles (Natsal-3)
Source: Sex Transm Infect. 2020 Mar 27;96(7):481–4. doi: 10.1136/sextrans-2019-054342 (PMC7591710; doi:10.1136/sextrans-2019-054342)
Supplement: Supplementary data [file sextrans-2019-054342supp001.pdf]

Supplementary Material

Title: The effect of weighting for sampling and non-response on estimates of sexually transmitted infection prevalence in the third British National Survey of Sexual Attitudes and Lifestyles (Natsal-3)

Supplementary Figure 1: Schematic of the sampling and response process, weights required to generate population-level prevalence measures and response rates in Natsal-3

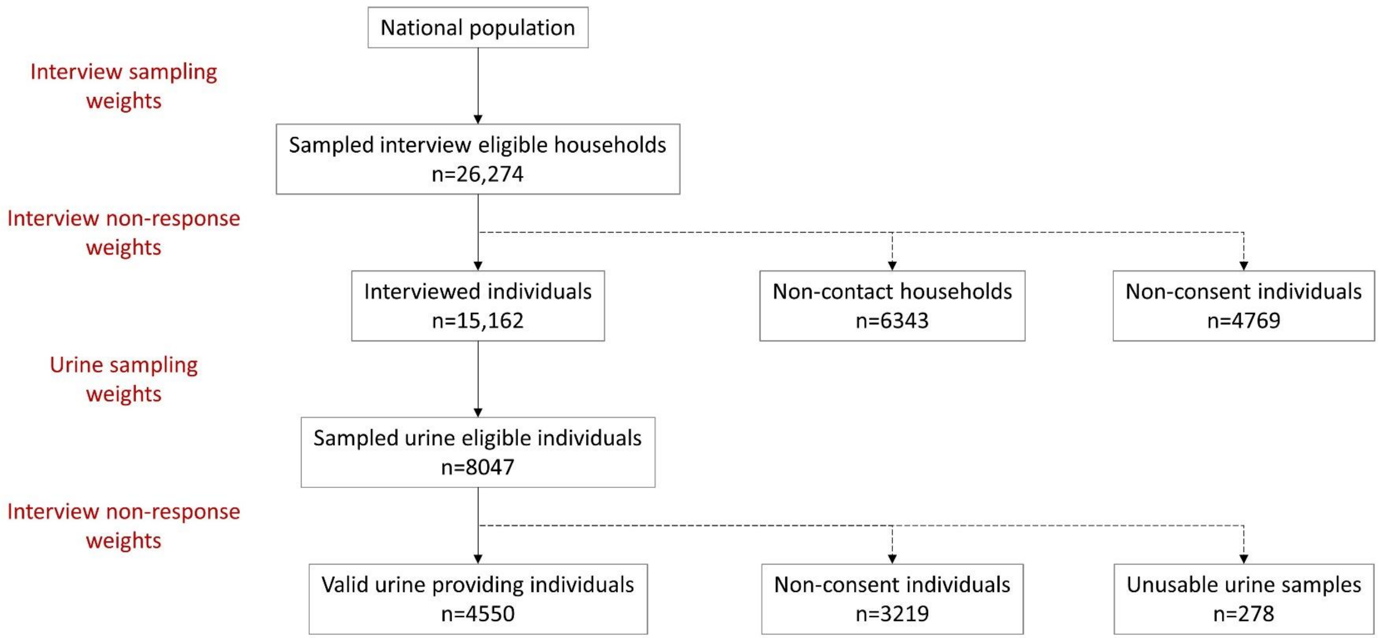

Reasons for non-contact included illness, inability to speak English or any other reason for not discussing participation with the respondent or a proxy. Non-consent included non-consent by the selected individual or their proxy. Overall non-response comprises non-contact, non-consent and unusable samples.

Supplementary Table 1: Relative impact of weighting components on Natsal-3 biomarker estimates

		Unweighted		Interview sample		+ interview non-response		+ urine sample		+ urine non-response	
Any oncogenic HPV	Female	20.4	[18.9 - 22.1]	17.1	[15.5 - 18.8]	17.5	[16.0 - 19.2]	17.1	[15.5 - 18.8]	15.9	[14.4 - 17.5]
Any Nonavalent HPV	Female	16.5	[15.1 - 18.0]	13.8	[12.4 - 15.3]	14.2	[12.8 - 15.8]	13.9	[12.5 - 15.4]	13.1	[11.7 - 14.6]
HPV types 6, 11, 16, 18	Female	9.0	[7.9 - 10.1]	7.5	[6.4 - 8.6]	7.6	[6.6 - 8.8]	7.4	[6.4 - 8.6]	7.0	[6.0 - 8.1]
HPV types 16, 18	Female	5.6	[4.8 - 6.6]	4.5	[3.7 - 5.5]	4.5	[3.7 - 5.5]	4.5	[3.7 - 5.4]	4.2	[3.4 - 5.2]
Chlamydia trachomatis	Female	2.33	[1.81 - 2.98]	1.59	[1.20 - 2.10]	1.63	[1.23 - 2.15]	1.55	[1.16 - 2.06]	1.46	[1.08 - 1.96]
	Male	1.91	[1.39 - 2.63]	1.34	[0.95 - 1.90]	1.26	[0.90 - 1.78]	1.20	[0.85 - 1.70]	1.08	[0.74 - 1.57]
Neisseria gonorrhoeae	Female	0.11	[0.04 - 0.35]	0.05	[0.02 - 0.15]	0.05	[0.02 - 0.16]	0.04	[0.01 - 0.14]	0.04	[0.01 - 0.11]
	Male	0.11	[0.03 - 0.42]	0.04	[0.01 - 0.17]	0.04	[0.01 - 0.15]	0.04	[0.01 - 0.14]	0.03	[0.01 - 0.14]
HIV	Female	0.12	[0.04 - 0.36]	0.11	[0.04 - 0.35]	0.10	[0.03 - 0.33]	0.11	[0.03 - 0.34]	0.12	[0.04 - 0.38]
	Male	0.32	[0.15 - 0.72]	0.25	[0.11 - 0.58]	0.26	[0.11 - 0.61]	0.25	[0.10 - 0.60]	0.22	[0.09 - 0.56]
Mycoplasma genitalium	Female	1.82	[1.37 - 2.42]	1.58	[1.09 - 2.29]	1.50	[1.05 - 2.14]	1.48	[1.03 - 2.13]	1.31	[0.91 - 1.88]
	Male	1.28	[0.86 - 1.90]	1.10	[0.70 - 1.74]	1.06	[0.68 - 1.65]	1.10	[0.70 - 1.71]	1.16	[0.73 - 1.85]
Trichomonas vaginalis	Female	0.27	[0.13 - 0.55]	1.34	[0.95 - 1.90]	1.26	[0.90 - 1.78]	1.20	[0.85 - 1.70]	1.08	[0.74 - 1.57]

Oncogenic HPV are types 16, 18, 31, 33, 35, 39, 45, 51, 52, 56, 58, 59 and 68 (i.e., Group 1 and Group 2A); Nonavalent types are 6, 11, 16, 18, 31, 33, 45, 52 and 58.
